# Supplementary material for: Divergent Cl- and H+ pathways underlie transport coupling and gating in CLC exchangers and channels
Source: eLife. 2020 Apr 28;9:e51224. doi: 10.7554/eLife.51224 (PMC7274781; doi:10.7554/eLife.51224)
Supplement: Supplementary file 1. — The following parameters from Boltzmann fits for CLC-7 and CLC-0 constructs used in Figures 5–7 are reported as mean ± S.E.M.: pmin, minimal open probability; V0.5, voltage of half maximal activation; z, gating charge; N, number of oocytes; n, number of independent oocyte batches. * indicates values of the fit parameters that were not well constrained during fitting, as such they should be considered as estimates of the parameters. [file elife-51224-supp1.docx]

| **Protein** | **cRNA** | **tRNA** | **p_min_** | **V_0.5_** | **z** | **N (n)** |
| --- | --- | --- | --- | --- | --- | --- |
| **CLC-7** |  | | | | | |
|  | F301A |  | 0.786 ± 0.001 | 56 ± 2 | 0.59 ± 0.02 | 18 (4) |
|  | F514A |  | 0.04 ± 0.01 | 41 ± 2 | 1.04 ± 0.04 | 12 (4) |
| **CLC-0** |  | | | | | |
| *single-pore gate* | WT |  | 0.000 ± 0.003 | -72 ± 3 | 0.71 ± 0.01 | 21 (10) |
|  | F214A |  | 0.62 ± 0.03* | -1 ± 12* | 0.46 ± 0.07* | 8 (2) |
|  | F418A |  | 0.69 ± 0.09* | -85 ± 34* | 0.49 ± 0.12* | 8 (3) |
|  | F214X | Phe | 0.00 ± 0.00 | -73 ± 4 | 0.62 ± 0.02 | 9 (4) |
|  | F214X | Cha | 0.03 ± 0.01 | -48 ± 2 | 0.84 ± 0.02 | 10 (3) |
|  | F214X | 2,6F_2_-Phe | 0.02 ± 0.01 | -53 ± 4 | 0.66 ± 0.02 | 9 (2) |
|  | F418X | Phe | 0.01 ± 0.01 | -76 ± 5 | 0.66 ± 0.05 | 11 (4) |
|  | F418X | Cha | 0.06 ± 0.01 | -25 ± 4 | 0.90 ± 0.02 | 11 (3) |
|  | F418X | 2,6F_2_-Phe | 0.00 ± 0.00 | -126 ± 3 | 0.67 ± 0.05 | 10 (3) |
|  | F418X | 2,6diMeth-Phe | 0.31 ± 0.02 | -33 ± 2 | 1.01 ± 0.04 | 9 (2) |
|  | C212S F418X | Phe | 0.00 ± 0.01 | -77 ± 4 | 0.64 ± 0.02 | 7 (2) |
|  | C212S F418X | 2,6diMeth-Phe | 0.07 ± 0.02 | -38 ± 2 | 0.87 ± 0.02 | 9 (3) |
|  |  | | | | | |
| *common-pore gate* | WT |  | 0.27 ± 0.02 | -93 ± 1 | 2.60 ± 0.10 | 19 (10) |
|  | F214A |  | 0.71 ± 0.04 | -53 ± 1 | 1.29 ± 0.10 | 9 (3) |
|  | F418A |  | 0.53 ± 0.02 | -86 ± 2 | 2.06 ± 0.16 | 12 (5) |
|  | F214X | Phe | 0.27 ± 0.04 | -92 ± 2 | 2.50 ± 0.17 | 7 (3) |
|  | F214X | Cha | 0.44 ± 0.04 | -79 ± 2 | 2.71 ± 0.11 | 9 (3) |
|  | F214X | 2,6F_2_-Phe | 0.23 ± 0.02 | -98 ± 2 | 2.54 ± 0.09 | 6 (2) |
|  | F418X | Phe | 0.24 ± 0.04 | -88 ± 2 | 2.49 ± 0.09 | 7 (3) |
|  | F418X | Cha | 0.55 ± 0.06 | -56 ± 4 | 2.14 ± 0.16 | 7 (3) |
|  | F418X | 2,6F_2_-Phe | 0.16 ± 0.03 | -108 ± 3 | 1.88 ± 0.08 | 5 (2) |
|  | F418X | 2,6diMeth-Phe | 0.07 ± 0.01 | -112 ± 4 | 2.03 ± 0.07 | 5 (2) |
